# Phonon Dominated Thermal Transport in Metallic Niobium Diselenide from First Principles Calculations

**DOI:** 10.3390/nano13020315

**Published:** 2023-01-12

**Authors:** René Contreras, Diego Celentano, Tengfei Luo, Zeyu Liu, J. O. Morales-Ferreiro

**Affiliations:** 1 Facultad de Ingeniería, Departamento de Tecnologías Industriales, Universidad de Talca, Camino Los Niches Km 1, Curicó 3340000, Chile; 2 Departamento de Ingeniería Mecánica y Metalúrgica, Centro de Investigación en Nanotecnología y Materiales Avanzados (CIEN-UC), Millennium Institute on Green Ammonia as Energy Vector (MIGA), Pontificia Universidad Católica de Chile, Av. Vicuña Mackenna 4860, Macúl, Santiago 8331150, Chile; 3 Department of Aerospace and Mechanical Engineering, University of Notre Dame, Notre Dame, IN 46556, USA; 4 Department of Chemical and Biomolecular Engineering, University of Notre Dame, Notre Dame, IN 46556, USA; 5 Department of Applied Physics, School of Physics and Electronics, Hunan University, Changsha 410082, China

**Keywords:** thermal conductivity, niobium diselenide, first-principles simulation, Boltzmann transport equation, thermoelectric

## Abstract

Niobium diselenide (NbSe_2_) is a layered transition metal dichalcogenide material which possesses unique electrical and superconducting properties for future nanodevices. While the superconducting, electrical, and bulk thermal transport properties of NbSe_2_ have been widely studied, the in-plane thermal transport property of NbSe_2_, which is important for potential thermoelectric applications, has not been thoroughly investigated. In this report, we study the lattice in-plane thermal transport of 2D NbSe_2_ by solving the phonon Boltzmann transport equation with the help of the first principles calculation. The thermal conductivity obtained at room temperature is 12.3 W/mK. A detailed analysis shows that the transverse acoustic phonon dominates the lattice thermal transport, and an anomalously small portion of electron contribution to the total thermal conductivity is observed for this metallic phase. The results agree well with experimental measurements and provide detailed mode-by-mode thermal conductivity contribution from different phonon modes. This study can provide useful information for integrating NbSe_2_ in nanodevices where both electrical and thermal properties are critical, showing great potential for integrating monolayer NbSe_2_ to thermoelectric devices.

## 1. Introduction

The discovery of two-dimensional (2D) materials, exemplified by graphene [1,2,3,4] and transition metal dichalcogenides (TMDCs) [5,6,7], has inspired significant research interests given their unique and superior structural, mechanical, optical, electrical, and thermal properties [8,9,10,11] showing great potential in photovoltaics [12,13], transistors [14,15], optoelectronics [16,17], sensing [6,18], and wearable heating and cooling [19]. Moreover, TMDC monolayers have shown great potential in nanoelectronics due to their intrinsic band gaps compared with graphene [6]. Early studies demonstrated niobium diselenide (NbSe_2_) to be among the first few layered-structured superconductors, with T*_c_* ranging from 5.9 to 7.0 K [20,21,22], showing great impact on both fundamental physics and possible industrial applications. The coexistence of the many-body collective charge density wave (CDW) and superconducting in NbSe_2_ has led to rich physics. For example, a much higher CDW transition temperature is achieved in the monolayer form compared with the bulk one [23], and a unique two-fold symmetry in superconducting can be experimentally observed in its monolayer form, showing an unconventional d-wave and p-wave coupling channel [24]. Additionally, the electron-phonon coupling is found to dominate the CDW transition process studied by the temperature dependent Raman experiments [22].

The thermal transport property, which is crucial for the reliability and performance of the electronic or spintronic devices is, however, less studied for niobium diselenide. As a potential thermoelectric candidate, the detailed information of thermal transport property and the ability of tunning this property also stand at the center stage, requiring further study from both experimental and computational angles. Similar to the case that the electronic structure [25,26], magnetic properties [27,28], and superconducting properties [21] can be quite different from their bulk form in TMDCs, unique features in thermal transport may exist in few or even monolayer NbSe_2_. Another challenge in experimentally determining the thermal transport properties in the few layer or monolayer NbSe_2_ is the tendency for oxidation under ambient environment [29], making high quality samples rare and the existing experimental results ambiguous. For example, recent thermoelectric properties studies of bulk NbSe_2_ showed across-plane thermal conductivity of 2.1 W/mK, and a recent study of transport properties of 4-layer NbSe_2_ in the low temperature range reported an in-plane thermal conductivity of 32 ± 10 W/mK at 200 K using a heat diffusion imaging method [30]. It also shows that the thermal conductivity can rise to a maximum of 53 ± 11 W/mK at 120 K, and then decrease as temperature increases. Along with this study, a value of 15 ± 4 W/mK at room temperature for the exfoliated 2H-NbSe_2_ flakes via a temperature dependent Raman spectroscopy was reported [31].

Considering the discrepancy among the reported thermal conductivity of monolayer NbSe_2_, the metallic nature of monolayer NbSe_2_ where both electrons and phonons can be the heat carriers, and the lack of mode-by-mode thermal transport contribution, a parameter-free lattice thermal transport study of NbSe_2_ is of great significance. This can illustrate detailed, mode-by-mode thermal conductivity analysis, providing useful insights for integrating monolayer NbSe_2_ in spintronics, nano-electronics, or thermoelectric devices.

## 2. Methods

In this work, we utilized the parameter-free first principles simulation based on the density functional theory (DFT) [32], and the linearized Boltzmann transport equation (BTE) was solved. A detailed review for this group of methods can be found in the reference [33]. The crystal structure of the monolayer niobium diselenide is first fully optimized, making atoms in the crystals force-free. The thermal conductivity then is calculated via an iterative solution of the phonon BTE using the ShengBTE package [34], with the help of the DFT based force constants calculated from the Quantum Espresso package [35]. Generalized gradient approximation (GGA) parameterized by Perdew, Burke, and Ernzerhof (PBE) is used as the exchange-correlation functional [36] in the DFT calculation, and the Vanderbit ultrasoft pseudopotential are used for both elements. A 50 Ry kinetic energy cutoff of the plane-wave basis functions is applied for all calculations, and the Brillouin zone is then discretized as a grid of 12 × 12 × 1 via the Monkhorst–Pack scheme [37]. A large z-direction vacuum space of 20 Å is left in the simulation box to prevent interactions between the layer and its periodic images in the cross-plane direction to simulate this monolayer. Our first principles optimized lattice parameters are calculated to be a = b = 3.474 Å, and it agrees well with the experimental values a = b = 3.445 Å [30]. The simulation is conducted on CPU, while the rapid evolution of DFT calculation on GPU may enable a much larger scale simulation, which is important for the thermoelectric device design.

After crystal structure relaxation, we then calculated the harmonic force constants using the density functional perturbation theory (DFPT) [38] implemented in Quantum Espresso with a first Brillouin Zone q-point grid size 8 × 8 × 1. A finite, difference-based method was used to calculate the cubic force constants via a 5 × 5 × 1 supercell of NbSe_2_ implemented in the thirdorder.py of ShengBTE [34]. A cutoff radius of 4 Å for third-order force constants was chosen for the calculation after a convergence test. The linearized phonon BTE was then solved in the ShengBTE [34] iteratively, considering the three-phonon scattering process with the help of the calculated force constants. Besides this popular open-source BTE solver, the transport properties were also verified in our in-house BTE solver [39], and the results agree. The BTE is solved in q-mesh of 30 × 30 × 1 of the first Brillouin zone.

**Figure 1:**
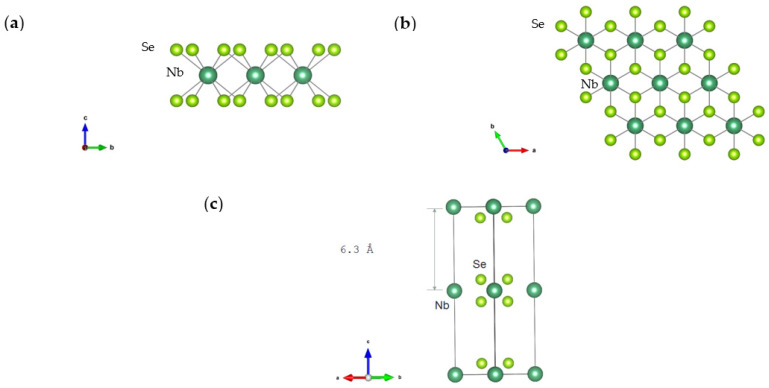
The crystal structure of NbSe_2_ in (**a**) Front view, (**b**) Upper view, (**c**) Side view if the bulk NbSe_2_ crystal structure to visualize the interlayer distance.

It should be emphasized that the choice of thickness in 2D materials can be somewhat arbitrary, and this choice can affect the reported values of various transport properties including thermoelectric, thermal, and electronic conductivity. [40] Considering that the thickness can be ill-defined in 2D materials, and the 3D intensive electrical and thermal conductivity requires a well-defined dimension independent description, the choice of thickness is important. In this work, a thickness of 6.3 Å (the interlayer distance of bulk NbSe_2_ crystal) is used for reporting results (Figure 1). A fair comparison of the thermal properties can be further derived using a common standard thickness, since the heat will only go through the one-layer structure, independent of how we defined the thickness or how ‘thick’ or ‘thin’ the structure [40,41,42] is. It is also necessary to note that the scattering beyond three-phonon scattering—for example, the four-phonon scattering process, is also of great importance [43,44]. Considering the relatively low thermal conductivity reported in experiments and the relatively low temperature, the impact of four-phonon scattering, as well as other effects such as the electron-phonon scattering, phonon renormalization, is out of scope of this research.

## 3. Results and Discussions

**Figure 2:**
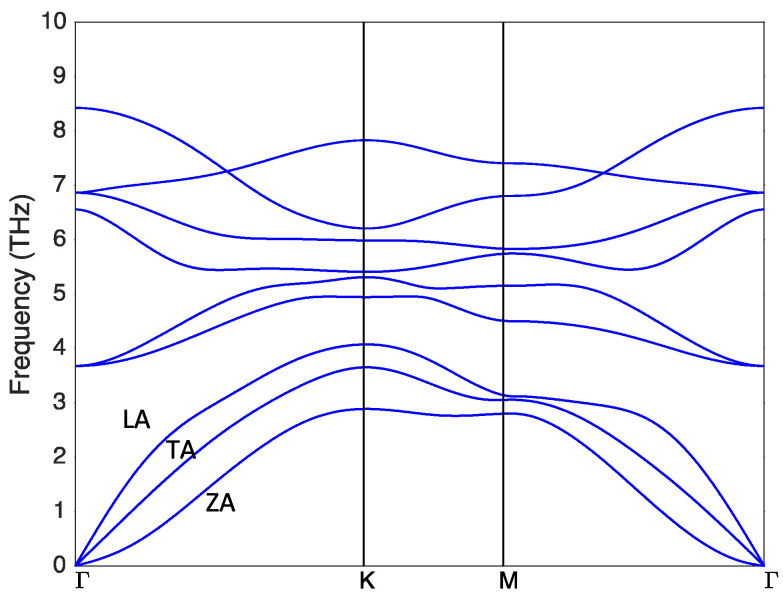
Phonon dispersion relation of the monolayer NbSe_2_. The three acoustic phonon modes are labeled.

We first visualized the phonon dispersion relation of the monolayer NbSe_2_ (Figure 2) shown in the high symmetric lines calculated via DFPT. From Figure 2, one unique feature is the absence of the complex cross-over session between the low frequency acoustic and higher frequency optical phonon modes in some other 2D materials [42], due to the structure symmetry, but a phonon ban gap is absent, indicating possible low thermal conductivity [45]. Quadratic flexural ZA phonon modes can be observed near the Gamma point, a common feature of the 2D materials [46,47,48]. This monolayer form of NbSe_2_ can be confirmed to be dynamically stable with no appearance of the imaginary phonon frequencies. For the convenience of the latter discussion, the other two acoustic phonon modes, namely the transverse acoustic (TA) and the longitudinal acoustic (LA) phonon, are also labeled in Figure 2.

**Figure 3:**
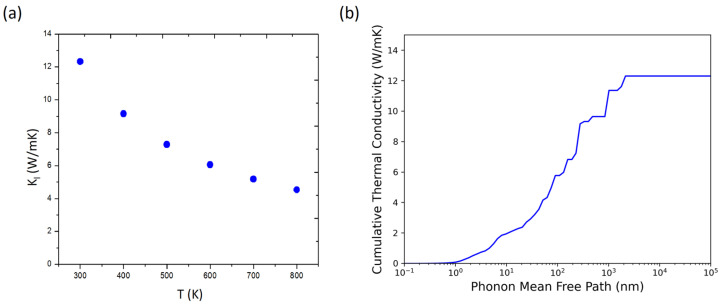
(**a**) The temperature dependent lattice thermal conductivity and (**b**) the cumulative thermal conductivity at 300K with respect to phonon MFP for the monolayer NbSe2.

The lattice thermal conductivity as a function of temperature and the cumulative thermal conductivity with respect to different phonon mean free path (MFP) cutoff of the monolayer NbSe_2_ is shown in Figure 3. Convergence tests of thermal conductivity with respect to the Brillouin zone grid size and third order force constants cutoff have been performed to make sure the fidelity of this calculation. We can report the calculated lattice thermal conductivity is 12.3 W/mK at room temperature using our thickness setup.

In Figure 3a, we can see a decreasing trend of the thermal conductivity with increasing temperature, a typical nature considering the anharmonic phonon scattering (Figure 3a) [49]. The scaling relation can be summarized as T^−1.01^ in NbSe_2_. This is extremely close to the typical fully anharmonic T^−1^ scaling in the high temperature regime, indicating the dominating Umklapp three-phonon scattering [50] in the scattering process and using monolayer NbSe_2_ as the platform for phonon hydrodynamics [51] can be fully fruitless. This calculated thermal conductivity is relatively low compares with other 2D materials for example graphene (3846 W/mK), black phosphorene (78 W/mK), blue phosphorene (130 W/mK), penta-graphene (368 W/mK), BN (1055 W/mK), MoS_2_ (34.5 W/mK), MoSe_2_ (54.21 W/mK), WS_2_ (141.9 W/mK), WSe_2_ (52.47 W/mK) and HfS_2_ (16.56 W/mK) [5,40,42,52]. Additionally, the cumulative thermal conductivity at room temperature with respect to different cutoffs of the MFP is shown in Figure 3b. It can be noted that the phonon thermal conductivity is mostly contributed by phonons with MFP greater than 10 nm. This can be a useful feature to improve the material for potential thermoelectric applications, since electron MFP are usually below 10 nm in 2D materials [52], and a nanostructure with a 10nm characteristic length may reduce thermal conductivity significantly while holding electric transport properties unchanged. Sure, thermal conductivity is not the only important property responsible for integrating of NbSe2 in nanodevices or thermoelectric devices. Detailed calculation on electron and phonon transport properties such as electronic conductivity and Seebeck coefficients are still advised via mesoscale transport approach in order to study this nano structure effect besides this cumulative thermal conductivity versus MFP picture.

**Figure 4:**
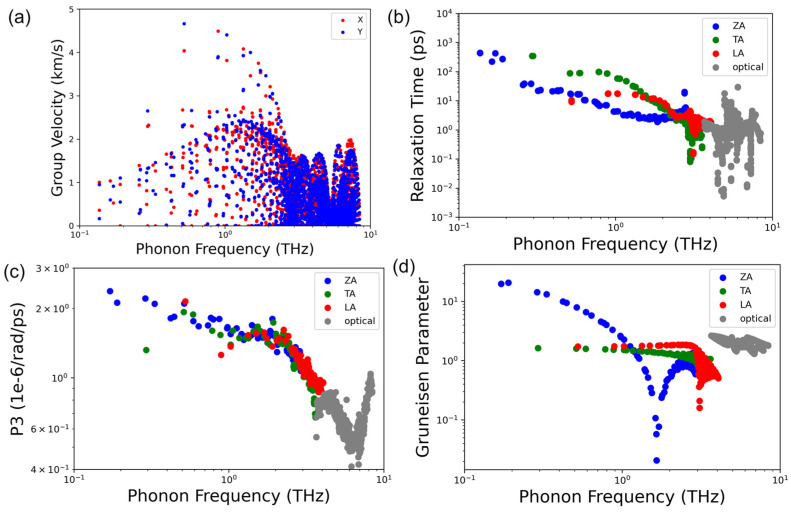
Scatter plots of (**a**) the phonon group velocity as a function of phonon frequency, (**b**) the phonon relaxation time, (**c**) the three-phonon scattering phase space (P3 in the figure) and (**d**) the Grüneisen parameters for different acoustic phonon modes as well as the optical phonon as a function of frequency at room temperature.

To understand how heat is transported by phonons in NbSe_2_, we first visualized the detailed group velocity profiles as a function of the phonon frequency in Figure 4a for different directions. It can be seen that the phonon group velocity in the X direction is similar to that in the Y direction, indicating the absence of anisotropy, agreeing with the crystal structure. One interesting feature in NbSe_2_ is the contribution of thermal conductivity from each polarization. The ZA, TA, and LA acoustic phonon modes contribute to the thermal conductivity with 2.80 W/mK, 6.74 W/mK, and 2.24 W/mK, respectively, and the optical modes combined contribute only 0.48 W/mK. It is clear that in NbSe_2_, the TA modes dominate the thermal transport in lattice. This TA mode domination is no stranger to lattice thermal transport in various 2D and 3D systems. Earlier pioneer work assumes that the entire heat is transported by the transverse phonons at high temperature [53]; in the 1970s and later quantitative work also proved that the TA phonon contributes significantly in Ge [54]. From the view of the well-known phonon relaxation time approximation (RTA), thermal conductivity can be modeled as $\kappa \propto \underset{qs}{\sum }C_{qs}v_{qs}^{2}\tau_{qs}$ [50,55], where $C_{qs}$, $v_{qs}^{2}$ and $\tau_{qs}$ are the specific heat, phonon group velocity, and the phonon relaxation time of the mode *qs*. It should be noted that a more complex iterative scheme is applied for thermal transport study here but considering the fact that the domination of the Umklapp scattering (T^−1.01^ temperature dependence), the analysis through this RTA is still useful and of high fidelity. Figure 4b shows the relaxation time as a functional of phonon frequency. We can see that the TA modes have larger relaxation time than that of the ZA and LA modes except for very low frequencies. This larger phonon lifetime can thus be considered as the primary reason for the dominate role of the TA modes in thermal transport, since neither group velocity nor the heat capacity of these modes are the highest of the three acoustic branches. (Obviously, the group velocity of TA modes is smaller than that of the LA mode viewed from the phonon dispersion relation in Figure 2).

In three-phonon scattering process there are two main aspects determining the phonon lifetime or relaxation time. One is the number of possible scattering channels and the other one is the strength of the scattering or how strong the scattering can be. The number of possible scattering channels can be quantified as the three-phonon scattering phase space (P3), counting the total number of possible scattering channels for certain phonon modes in the first Brillouin zone where energy and quasi-momentum conservation is satisfied [56]. The larger the phase space is, the more possible it is for this phonon mode to be scattered in either absorption or emission process. The other is the three-phonon scattering strength. The Grüneisen parameter is a physical parameter describing the anharmonicity of the materials for each phonon modes and thus commonly describing the strength of the three-phonon scattering [57,58]. A larger Grüneisen parameter means larger anharmonicity, leading to a stronger scattering and smaller thermal conductivity. It can be concluded in Figure 4c that the phonon scattering phase space of these three acoustic modes do not differ significantly, indicating that this scattering channel difference is not the main contributor to the large difference in thermal conductivity contributions. On the other hand, however, a clear difference of the Grüneisen parameters for these three acoustic phonon modes can be viewed in Figure 4d. In the long wave limit, the main contributor for thermal conductivity, ZA modes have a much larger anharmonicity and the TA phonons lead to the smallest anharmonicity, indicating this clear difference in the Grüneisen parameter is the main reason for the different contributions to thermal conductivity among the three acoustic phonon modes.

For this metallic phase, if we consider existing experimental works [31] where researchers measured the thermal conductivity, including contributions from both the phonons and electrons, we may extract the electronic thermal conductivity to be 15.0 − 12.3 = 2.7 W/mK if we take the electrical conductivity of bulk NbSe_2_ $\left(15\times 10^{-5}\;\Omega \cdot \mathrm{cm}\right)$ from the literature and calculate the corresponding electronic thermal conductivity using Wiedemann–Franz law (a value of 4.9 W/mK is obtained). Considering the uncertainty in the experimental measurement and quality of the sample, we deem the agreement between the calculations and experiments to be reasonable. Moreover, in the experiment, samples of thicknesses of 20, 25 and 120 nm were studied. These samples may consist of 32 layers or more of NbSe_2_, which may hardly be considered as the monolayer. Studies have shown that from single layer to bulk MoS_2_, a material similar to NbSe_2_ in structure and interlayer bonding the thermal conductivity can be reduced by ∼30% [48]. If we consider a similar reduction ratio for NbSe_2_, the lattice thermal conductivity of bulk NbSe_2_ would be 8.6 W/mK. To summarize, with the electronic thermal conductivity from the Widemann-Franz law, a total thermal conductivity of 13.5 W/mK is obtained which is well within the uncertainty of the experimental result of k = 14 ± 5 W/mK.

Overall, this is an interesting finding for the metallic monolayer NbSe_2_, where the electron plays a smaller role in thermal transport compared to phonons. Intuitively, electron is the main heat carrier for metals due to the free carriers, and the phonon contribution is usually much smaller than that of electron. However, this is not the case in NbSe_2_. This may be understood as the effect of the relatively strong electron-phonon coupling in NbSe_2,_ which is critical for its superconductivity [22,23,59]. This phonon domination can be even larger if we consider a temperature dependent Lorenz ratio in various metals where a slightly smaller than unity Lorenz ratio can be found considering the full first-principles electron-phonon scattering [60,61,62].

## 4. Conclusions

In summary, a parameter free lattice thermal conductivity study of the monolayer NbSe_2_ was conducted using the first-principles phonon BTE. The lattice thermal conductivity of NbSe_2_ was calculated to be 12.3 W/mK at room temperature, agreeing well with existing experimental measurements. Detailed analysis shows that the transverse acoustic modes have the most significant contribution to total thermal conductivity. Viewed from the Grüneisen parameters, this TA phonon domination can be attributed to the small anharmonicity associated. It also finds an anomaly; a small contribution of electrons to the total thermal transport for this metallic phase, which can be traced to the strong electron-phonon coupling for this superconductor. This work illustrates detailed mode-by-mode thermal conductivity analysis, providing useful information for integrating NbSe_2_ in nanodevices or thermoelectric devices where both electrical and thermal properties are important.

## Data Availability

The data that support the findings of this study are available from the corresponding authors upon reasonable request.
